# Interactional Effects of Multidimensional Perfectionism and Cognitive Emotion Regulation Strategies on Eating Disorder Symptoms in Female College Students

**DOI:** 10.3390/brainsci11111374

**Published:** 2021-10-20

**Authors:** Germaine Y. Q. Tng, Hwajin Yang

**Affiliations:** School of Social Sciences, Singapore Management University, Level 4, 90 Stamford Road, Singapore 178903, Singapore; hjyang@smu.edu.sg

**Keywords:** socially prescribed perfectionism, cognitive emotion regulation, rumination, catastrophizing, self-blame, eating disorders

## Abstract

Given the inconclusive findings regarding the relation between perfectionism and eating disorder symptoms, it is important that we determine whether this relation is modulated by emotion dysregulation, which is a prominent risk factor for eating disorders. We sought to identify specific cognitive emotion regulatory strategies—rumination, self-blame, and catastrophizing—that interact with multidimensional perfectionism to shape eating disorder symptoms (i.e., shape, weight, eating concerns, and dietary restraint). Using latent moderated structural equation modeling, we analyzed data from 167 healthy young female adults. We found that only rumination significantly moderated the relation between socially prescribed perfectionism and eating disorder symptoms. However, this was not observed for self-oriented perfectionism or other regulatory strategies. These findings held true when a host of covariates were controlled for. Our findings underscore the crucial role of rumination, a modifiable emotion regulatory strategy, in augmenting the relation between socially prescribed perfectionism and eating disorder symptoms in young women.

## 1. Introduction

A substantial proportion of college-aged women (38.9%) [1] report subclinical eating disorder symptoms, including attitudinal (preoccupation with eating, shape, and weight concerns) and behavioral (e.g., dietary restraint and excessive exercise) disturbances [1,2]. In light of their psychological and physiological repercussions, understanding the risk factors for eating disorders allows us to intervene in potential diagnostic progression from subthreshold to full-threshold symptoms [3]. In this regard, cognitive-behavioral models of eating disorders have identified clinical perfectionism—an “overdependence of self-evaluation on the determined pursuit of personally demanding, self-imposed standards in at least one salient domain, despite adverse consequences” [4] (p. 778)—as a central risk factor for eating disorder symptoms [4,5]. For instance, the failure to meet perfectionistic standards in domains including weight and/or body shape [6] can foster counterproductive behaviors and self-criticism that manifest as symptoms of eating disorders [6,7,8]. It is notable, however, that potential moderators may qualify the impact of perfectionism on eating disorder symptoms. Given that perfectionism is a nonspecific risk factor that is elevated across multiple psychopathologies [7], some evidence indicates that perfectionism alone fails to sufficiently and independently explain individual differences in eating disorder symptomatology [9,10] and eating-related clinical impairment in young women [11]. This calls attention to the importance of examining other risk factors that may augment the impact of perfectionism to confer greater risk for eating disorder symptoms [12]. To this end, cognitive-behavioral [13] and socio-emotional [14] models of eating disorders highlight the role of emotion regulation (i.e., the processes responsible for monitoring, evaluating, and modifying emotional reactions [15] (p. 27)), particularly in reference to dysregulation or difficulties coping with adverse affective states. Supporting this, a growing body of studies has demonstrated that eating disorder symptoms in young women are concomitant with certain cognitive emotion regulatory strategies, i.e., cognitive processes that regulate the magnitude and/or type of aversive emotional experiences by reconstructing the meaning of negative situations [16]. Specifically, numerous lines of evidence suggest that eating disorder symptoms are linked to the use of maladaptive strategies, such as rumination, catastrophizing, and self-blame, that are characterized by persevering thoughts or self-criticism [17,18,19,20,21,22]. For instance, several meta-analyses [18,19,23] have elucidated relations between eating disorder symptoms and elevated levels of rumination. In particular, Meule et al. [18] found that individuals with restrictive anorexia nervosa and bulimia nervosa report greater use of dysfunctional cognitive regulatory strategies (i.e., self-blame, rumination, and catastrophizing) than healthy controls. Hence, it is plausible that young adults who have high levels of perfectionism and ineffectively regulate aversive emotions are at heightened risk for eating disorder symptoms and clinical impairment [11,24]. Building on cognitive-behavioral theories of eating disorders, therefore, we sought to delineate specific dimensions of perfectionism and cognitive emotion regulatory strategies that interact to predict eating disorder symptoms.

### 1.1. Limitations of Previous Studies

Although a handful of studies have focused on the relations among perfectionism, dysfunctional emotion regulatory strategies, and various facets of subclinical/clinical disordered eating, they are limited in three major respects. First, it is unclear which specific dimension(s) of perfectionism [25,26] is (are) associated with subclinical/clinical eating disorder symptoms. According to Hewitt and Flett (1991), dimensions of perfectionism can be delineated based on the perceived source of perfectionistic standards. Specifically, self-oriented perfectionism is the evaluation of oneself against self-imposed standards, whereas socially prescribed perfectionism consists of self-evaluation based on standards prescribed by significant others in one’s social environment. Previous findings are somewhat equivocal regarding the source of perfectionistic standards that foster eating disorders. For instance, several studies suggest that self-oriented perfectionism predicts significant variance in dietary restraint and the attitudes associated with eating disorders (e.g., [27,28]). However, some identify only socially prescribed perfectionism as a predictor of anorexic eating attitudes, such as fear of weight gain (e.g., [29,30]). In contrast, other studies suggest both dimensions of perfectionism as risk factors for the development of eating disorder symptoms—particularly dietary restraint and anorectic attitudes—in healthy college-aged women (e.g., [27,31,32]). Thus, to disentangle these mixed findings in the literature, it is essential that we simultaneously evaluate whether self-oriented and socially prescribed perfectionism are differentially related to eating disorder symptoms in female college students.

A second notable limitation of prior studies is that while emotion dysregulation has been suggested as a risk factor for eating disorder symptoms, the specific regulatory strategies that may elevate these symptoms in nonclinical samples have not been clearly identified. Prior studies on emotion dysregulation and eating disorder symptoms have predominantly conceptualized emotion dysregulation as broad difficulties related to the perception of emotional experiences, flexible strategy use, and tolerance of aversive emotions [33]. For instance, Haynos et al. [34] demonstrated that restrictive eating in healthy undergraduates was linked to heightened emotion regulation difficulties, including limited access to adaptive emotion regulation skills (see also [35,36]). Although this conceptualization sheds light on relevant difficulties in emotion regulation, it is critical that we understand the role of specific regulatory strategies. In light of this, Garnefski and Kraaij’s (2007) framework of cognitive emotion regulation conceptualizes nine regulatory strategies that are used in response to aversive emotions, a subset of which are considered maladaptive and have been identified as correlates or risk factors for eating disorder symptoms. Specifically, the link between eating disorder symptoms and rumination has been extensively supported (e.g., [21,37,38]), while emerging evidence indicates that levels of catastrophizing [22] and self-blame [17,39] are systematically related to the severity of eating disorder symptoms and likelihood of eating disorder remission. Therefore, to deepen our insight into potential interactions between crucial psychosocial factors for eating disorder symptoms, we sought to examine whether specific maladaptive emotion regulation strategies would interact with multidimensional perfectionism (self-oriented and socially prescribed) to predict eating disorder symptoms in healthy college-aged women. We focused on three major maladaptive strategies—rumination, catastrophizing, and self-blame—since their empirical importance has received growing attention in relation to eating disorder symptoms [17,21,22].

A third limitation of previous studies is their frequent use of manifest (observed) variables (e.g., [11,24]), which overlooks potential measurement errors that can obscure genuine interactive relations. For instance, inconsistent findings regarding the relation between multidimensional perfectionism and eating disorder symptoms (e.g., [40,41]) could stem from random measurement errors inherent to manifest variables. Conceivably, these errors may have similarly affected the true interaction effects of perfectionism and emotion regulation on eating disorder symptoms. Thus, we employed a more rigorous methodology, latent moderated structural equation modeling [42,43], which uses latent variables based on the common variance extracted from multiple indicators and, therefore, provides more unbiased estimates of the interaction between multidimensional perfectionism and emotion regulation strategies on eating disorder symptoms [44,45].

### 1.2. Present Study

Our research goals were three-fold. First, drawing on Hewitt and Flett’s (1991) multidimensional conceptualization of perfectionism, we sought to identify the specific dimension(s) of perfectionism that predict(s) eating disorder symptoms in college-aged women. Socially prescribed perfectionism involves the belief that self-worth is based on the attainment of externally imposed standards, while self-oriented perfectionism involves the belief that self-worth is based on the attainment of exceedingly high self-imposed standards [46,47]. Given that both self-oriented and socially prescribed perfectionism are characterized by a conditional sense of self-worth that is overly dependent on attaining high standards imposed by either the self or close others [47], we hypothesized that higher levels of self-oriented and socially prescribed perfectionism would independently serve as risk factors for eating disorder symptoms.

Second, we aimed to examine whether maladaptive emotion regulation strategies [48] magnify the relation between these perfectionism dimensions and eating disorder symptoms. In light of Blackburn et al.’s (2020) work on the intrapsychic experiences of anorexia nervosa, we hypothesized that specific maladaptive regulatory strategies (i.e., rumination, catastrophizing, self-blame) would escalate perfectionistic cognitions and feelings of inadequacy (stemming from perfectionistic standards), and thereby foster eating disorder symptoms to derive a sense of control or impose self-punishment.

Specifically, ruminative processes include the persistence and exacerbation of negative perfectionistic thoughts, such as overgeneralizing failures [49]. Moreover, ruminative tendencies have been shown to be associated with attitudinal and behavioral eating disorder symptoms in nonclinical samples (e.g., [37]). Drawing on intrapsychic processes involved in disordered eating [50], ruminative tendencies (i.e., repetitive, persevering thoughts about one’s inadequacies) are hypothesized to prolong and exacerbate adverse thoughts and emotions that arise when individuals with high levels of self-oriented or socially prescribed perfectionism fail to fulfil self- or other-imposed standards. As a result, eating disorder symptoms such as dietary restraint or weight management may serve as a means to regain a sense of control over objective metrics (e.g., calories and weight) and compensate for feelings of helplessness and inferiority [51].

Similarly, given that catastrophizing involves the overestimation of threatening outcomes related to one’s shortcomings, we hypothesized that perfectionistic individuals who catastrophize about failure-related consequences (e.g., “If I exceed my ideal body weight, I will be repulsive”) would report a greater degree of eating disorder symptoms (e.g., weight restriction). This is likely because eating disorder symptoms may temporarily provide a sense of reliability and control by helping to regulate catastrophic thoughts/emotions associated with anticipated failure-related consequences [52]. Consistently, individuals with eating disorders have been shown to display higher levels of catastrophic worry when compared to healthy controls [22].

Regarding self-blaming, we hypothesized that self-blaming for falling short of perfectionistic standards (e.g., ideal weight or shape) would exacerbate eating disorder symptomatology. That is, perfectionistic individuals who ascribe shortcomings to themselves (e.g., “It is my fault I’m not good enough”) are more likely to display eating disorder symptoms such as restrictive eating and desire for weight loss as a form of self-punishment [50]. Supporting this notion, attributional biases to ascribe failures to oneself (e.g., “I must be perfect and when I am not, it must be my fault” [53] (p. 351)) have been linked to eating disorder symptoms in women (e.g., [54]). In sum, these maladaptive cognitive strategies likely magnify or prolong perfectionistic cognitions about perceived discrepancies from unrealistic ideals, such that perfectionism would more strongly predict eating disorder symptoms in young women to the extent that they employ these maladaptive emotion regulatory strategies.

Third, we aimed to conduct a series of latent moderated structural equation modeling analyses to identify specific cognitive regulatory strategies [48] that moderate the pathways from distinct dimensions of perfectionism [25,26] to eating disorder symptoms, while controlling for a host of demographic (i.e., age, body mass index, and household income), affective (i.e., negative affectivity, depressive symptoms, and anxiety symptoms), and personality (i.e., neuroticism and conscientiousness) covariates that have been shown to influence or have shared risk factors with cognitive-behavioral eating disorder symptoms [55,56,57,58]. We hypothesized that self-oriented and socially prescribed perfectionism would predict a higher degree of eating disorder symptoms, especially for individuals who rely on rumination, catastrophizing, and self-blame as regulatory strategies. To examine our hypotheses, we focused on college-aged women because of their elevated rates of eating disorder symptomatology [59] and heightened vulnerability to eating disorder symptoms during this age window [60]. By elucidating the interactions between specific risk factors for eating disorder symptoms, we sought to advance the theoretical understanding and inform intervention strategies by accounting for multiple modifiable psychosocial factors.

## 2. Methods

### 2.1. Participants

A total of 167 female students aged 18 to 26 (*M_age_* = 21.60 years; *SD* = 0.52; *M_BMI_* = 20.69; *SD* = 2.56) from the social sciences faculty (comprising Psychology, Sociology, and Political Science majors) of a local university were recruited in exchange for course credit or monetary compensation ($10). Our sample size was deemed appropriate, since a minimum sample size of 156 is required for a structural equation model with a maximum of three latent variables and 10 manifest variables [61]. All participants gave their informed consent (see Table 1 for descriptive statistics).

### 2.2. Measures

#### 2.2.1. Multidimensional Perfectionism

The Multidimensional Perfectionism Scale (MPS-H [26]) was used to assess participants’ perfectionistic traits on a seven-point scale (1 = *strongly disagree*; 7 = *strongly agree*). Two 15-item subscales were selected to measure (a) self-oriented perfectionism (e.g., “One of my goals is to be perfect in everything I do,” α = 0.88) and (b) socially prescribed perfectionism (e.g., “My family expects me to be perfect,” α = 0.77), respectively. Higher scores denoted a greater level of each perfectionism dimension [26].

#### 2.2.2. Cognitive Emotion Regulation Strategies

Three subscales that assess the use of maladaptive cognitive emotion regulation strategies—self-blame (α = 0.80), rumination (α = 0.68), and catastrophizing (α = 0.81)—were selected from the Cognitive Emotion Regulation Questionnaire (CERQ [48]). Using a five-point scale (1 = *almost never*; 5 = *almost always*), participants rated their frequency of employing the respective regulatory strategies.

#### 2.2.3. Disordered Eating Symptoms

Attitudinal and behavioral eating disorder symptoms within the past 28 days were assessed using the Eating Disorder Examination Questionnaire-Global (EDE-Q [62]). The scale comprises four subscales: eating concerns (e.g., preoccupation with food, α = 0.78), shape concerns (e.g., discomfort from seeing one’s body, α = 0.86), weight concerns (e.g., fear of weight gain, α = 0.84), and restraining behaviors (e.g., following strict food rules, α = 0.82), which represent the attitudinal and behavioral symptoms associated with eating disorders [63]. Participants rated each item on seven-point scales with varying anchors, wherein higher scores denoted a greater degree of symptoms.

#### 2.2.4. Negative Affect

The 10-item Negative Affect subscale of the Positive and Negative Affect Schedule (α = 0.90 [64]) was used to measure negative affectivity. Participants rated the extent to which they had experienced specific emotions within the past week using a five-point Likert scale (1 = *not at all*; 5 = *extremely*). Relevant item scores were summed, with higher scores indicating greater negative affect.

#### 2.2.5. Depression and Anxiety

Depression and anxiety symptoms were assessed with their respective seven-item subscales from the Depression, Anxiety and Stress Scale (DASS-21 [65]). On a four-point rating scale (0 = *did not apply to me at all*; 3 = *applied to me very much*), participants rated the extent to which they had experienced symptoms of depression (α = 0.89; e.g., “I couldn’t seem to experience any positive feeling at all”) and anxiety (α = 0.87; e.g., “I felt I was close to panic”) over the past week.

#### 2.2.6. Personality Traits

Personality traits (i.e., neuroticism and conscientiousness) relevant to eating disorder symptoms were assessed as covariates using corresponding subscales from the Big Five Inventory [66,67,68]. The neuroticism subscale (α = 0.82; e.g., “I see myself as someone who worries a lot”) consists of eight items and the conscientiousness subscale (α = 0.71; e.g., “I see myself as someone who worries a lot”) nine items. Participants indicated their agreement with each statement on a five-point Likert scale (1 = *strongly disagree*; 5 = *strongly agree*). Higher subscale scores indicated higher levels of neuroticism and conscientiousness, respectively.

#### 2.2.7. Demographics

Age, socioeconomic status (SES), based on combined monthly household income, and body mass index (BMI) served as demographic covariates. Participants’ BMI, which is a widely used index of body fat that predicts symptoms of eating disorders [69], was calculated using the standard formula (weight in kg/[height in meters]^2^). SES has also been linked to unhealthy eating outcomes, although this is equivocal (see [70], for a review).

### 2.3. Procedure

This study was conducted within a two-week period from late February to early March. Participants completed the measures in this order: MPS-H (perfectionism), CERQ (cognitive emotion regulation strategies), EDE-Q (eating disorder symptoms), and questionnaires reporting negative affect, depression and anxiety symptoms, personality traits, and demographic information. All measures were administered online via computers in a laboratory. Upon completion, participants received course credit or a monetary reward ($10) and were thanked and debriefed. The study’s materials and procedures were approved by the university’s institutional review board (IRB-19-001-A030 (319)).

### 2.4. Analysis Plan

All analyses were conducted on *Mplus* 7.4 [71] using full information maximum likelihood estimation. Socially prescribed and self-oriented perfectionism were modeled as exogenous latent variables by parceling their respective subscale items as indicators [72]. We used parceling because it is well suited for unidimensional scales and has been shown to offer psychometric advantages, including enhancement of scale communality and reduction of random error [73]. The latent variables of rumination, catastrophizing, and self-blame were each specified by their four respective subscale items as indicators. Eating disorder symptoms were modeled as an endogenous latent variable using the four EDE-Q subscale scores as indicators: weight-restricting behaviors (e.g., dietary restraint and rule-following), shape concerns (e.g., preoccupation with body shape), weight concerns (e.g., fear of weight gain), and eating concerns (e.g., guilt about eating).

To ascertain that the indicators adequately reflected their intended constructs, the model fit of each measurement model was examined through confirmatory factor analysis based on the following criteria: normed chi-square values ($\frac{\chi^{2}}{df})$ below 2, comparative fit index (CFI) and Tucker-Lewis Index (TLI) values ≥ 0.95, standardized root-mean-square residual (SRMR) values ≤ 0.08, and root-mean-square error of approximation (RMSEA) values ≤ 0.08 and ≤ 0.06 to denote acceptable and good fit, respectively [74,75]. All reported estimates were standardized. For latent moderated structural equation modeling, we followed a two-step estimation approach [42,43] to examine the interactional effects of multidimensional perfectionism and each cognitive emotion regulation strategy on eating disorder symptoms. For each latent moderation analysis, a structural model (i.e., baseline model) was first estimated without the latent interaction term to evaluate its fit to the data. Thereafter, when the latent interaction term was added to the model (i.e., the alternate model), we assessed the difference in model fit between the two models with a log-likelihood ratio test (i.e., Δ-2LL test [42]), since conventional fit indices (i.e., CFI, TLI, RMSEA, SRMR, and χ^2^) are not provided under latent moderated structural modeling. A significant difference in the log-likelihood ratio, based on a chi-square distribution test, indicates that the model with an interaction term explains the data better than the one without an interaction term [76]. In all analyses, demographic (age, BMI, and socioeconomic status), affective (negative affect, depressive symptoms, and anxiety symptoms), and personality (neuroticism and conscientiousness) covariates were controlled for (see Table 2 for zero-order correlations between all variables).

## 3. Results

### 3.1. Measurement Models

First, we performed a series of confirmatory factor analyses to ascertain the fit of each measurement model. All individual measurement models showed excellent fit (see Table 3 for all fit indices). Next, we assessed the adequacy of the full-measurement models, which included the latent factors of perfectionism, eating disorder symptoms, and their respective regulatory strategies. The full-measurement model for socially prescribed perfectionism (as a focal predictor) and rumination showed an excellent fit, while the other two full-measurement models—which included self-blame and catastrophizing, respectively—showed acceptable fit. Separately, the full-measurement model for self-oriented perfectionism (as a focal predictor) and rumination demonstrated excellent fit, whereas the other full-measurement models—which included self-blame and catastrophizing, respectively—showed acceptable fit. In all measurement models, all factor loadings were significant (*ps* ≤ 0.003). Moreover, correlating the residuals of two items with respect to the rumination (2nd and 3rd), catastrophizing (2nd and 4th), and self-blame (3rd and 4th) subscales significantly improved the fit of their corresponding full-measurement models, because they were similarly worded to measure preoccupation with stressful experiences, thoughts about a frightening experience, and thoughts about one’s mistakes causing stressful life events, respectively [16].

### 3.2. Latent Moderated Structural Equation Analyses

We examined two sets of latent moderated structural equation models with socially prescribed perfectionism and self-oriented perfectionism as focal predictors, respectively, while controlling for demographic, affective, and personality covariates. Each set of models included one of the three potential moderators in turn—i.e., rumination, catastrophizing, and self-blame strategies.

#### 3.2.1. Socially Prescribed Perfectionism

**Figure 1:**
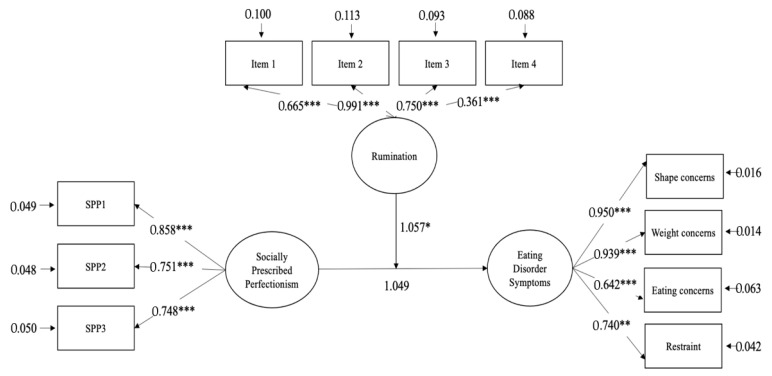
Adjusted structural equation model with rumination as a moderator for the pathway between socially prescribed perfectionism and disordered eating. Circles represent latent factors and squares represent indicators. Covariates are not depicted for brevity. Values on the longer, single-headed arrows signify path coefficients. Values for the smaller, single-headed arrows represent residual variances. All coefficients shown are standardized and obtained statistical significance at the 0.05 level. * *p* < 0.05; ** *p* < 0.01; *** *p* < 0.001.

**Figure 2:**
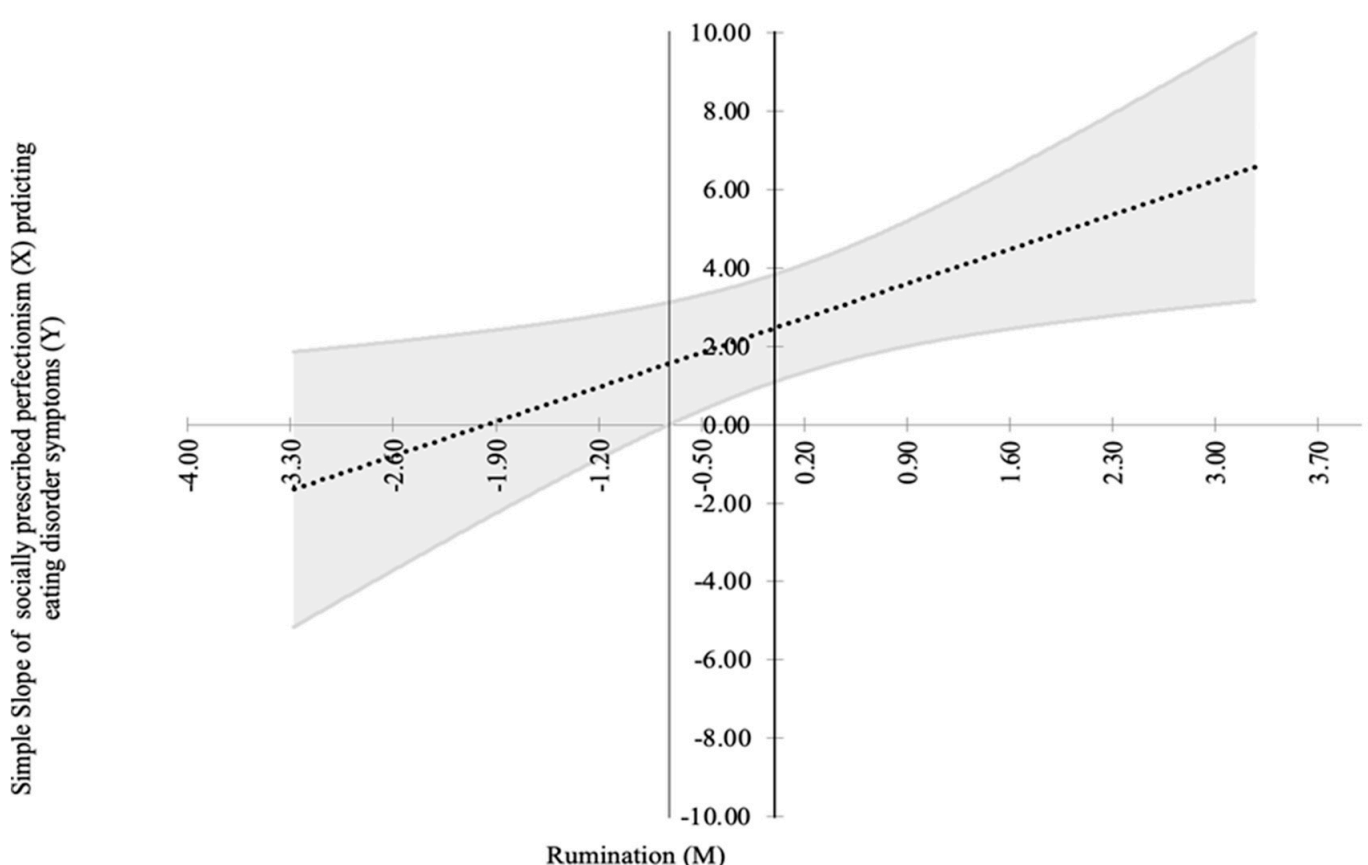
This Johnson-Neyman plot illustrates the simple slope of socially prescribed perfectionism that predicts disordered eating at varying degrees of rumination (unadjusted model). Socially prescribed perfectionism and rumination are mean-centered. The dash-dot line denotes the simple slope with 95% confidence bands. The positive relation between socially prescribed perfectionism and disordered eating is significant only at higher values of rumination.

***Rumination***. The baseline model without an interaction term fit the data fairly well. When the socially prescribed perfectionism × rumination interaction term was added to the baseline model, we found a significant interaction effect, *B* = 1.057, SE = 0.508, 95% CI [0.062, 2.053] (see Figure 1). This indicates that rumination significantly moderated the relation between socially prescribed perfectionism and eating disorder symptoms. When a log-likelihood ratio (Δ-2LL) test was performed to compare the models with and without an interaction term, the baseline model without an interaction term did not show a significant loss of model fit compared with the alternate model with an interaction effect, χ^2^ (1) = 0.45, *p* = 0.502, which suggests that the alternate model did not better explain the data. When we probed the significant moderation effect using the Johnson-Neyman approach, we found that the positive relation between socially prescribed perfectionism and eating disorder symptomatology was significant at higher (i.e., at least −0.38 *SD* or the latent factor value of −0.4113), but not lower, levels of rumination (see Figure 2).

In order to examine if the interaction effect between socially prescribed perfectionism and rumination uniquely predicts different facets of eating disorder symptoms, we performed further analyses to delineate between weight concerns, shape concerns, eating concerns, dietary restraint, and binge-compensatory behaviors (i.e., overeating, self-induced vomiting, laxative consumption, and compensatory exercise). Controlling for all covariates, we found that the socially prescribed perfectionism × rumination interaction effect only explained dietary restraint (*B* = 0.591, SE = 0.293, 95% CI [0.017, 1.166]) and weight concerns (*B* = 0.901, SE = 0.372, 95% CI [0.171, 1.631]), but did not significantly predict eating concerns (*B* = 0.207, SE = 0.281, 95% CI [−0.343, 0.757]), shape concerns (*B* = 0.892, SE = 0.516, 95% CI [−0.121, 1.904]), or binge-compensatory behaviors (*B* = 0.814, SE = 1.484, 95% CI [−3.723, 2.095]).

Further exploratory analyses were conducted to examine whether the socially prescribed perfectionism × rumination interaction effect similarly explained depressive and/or anxiety symptoms. We found that the socially prescribed perfectionism × rumination interaction term did not predict symptoms of depression (*B* = 0.114, SE = 0.119, 95% CI [−0.119, 0.348]) or anxiety (*B* = 0.077, SE = 0.085, 95% CI [−0.090, 0.244]) when demographic covariates (i.e., age, BMI, and socioeconomic status) were accounted for. These findings, therefore, lend support to the specificity of the observed interaction effect to eating disorder symptoms.

***Catastrophizing and self-blame***. We found that socially prescribed perfectionism did not interact with catastrophizing (*B* = −1.583, SE = 0.987, 95% CI [−3.419, 0.252]) or self-blame (*B* = 2.444, SE = 1.407, 95% CI [−0.314, 5.203]) to predict eating disorder symptoms. Furthermore, log-likelihood ratio (Δ-2LL) tests showed that their respective baseline models did not show significant loss in fit, and thus, suggest that baseline models were more parsimonious than their corresponding interaction models.

Across all baseline models, socially prescribed perfectionism failed to independently predict eating disorder symptoms when rumination (*B_baseline_* = 0.142, SE = 0.102, 95% CI [−0.428, 2.323]), catastrophizing (*B_baseline_* = 0.109, SE = 0.108, 95% CI [−0.691, 2.086]), or self-blame (*B_baseline_* = 0.129, SE = 0.098, 95% CI [−0.448, 2.132]) were included as predictors. Moreover, in these baseline models, only catastrophizing predicted eating disorder symptoms (*B_catastrophizing_* = 0.216, SE = 0.101, CI [0.113, 4.261]), while rumination (*B_rumination_* = 0.015, SE = 0.088, CI [−1.373, 1.639]) and self-blame did not (*B_self-blame_* = −0.098, SE = 0.062, CI [−7.537, 0.942]). Across all alternate models, we found that socially prescribed perfectionism again failed to predict eating disorder symptoms when rumination (*B_alternate_* = 1.049, SE = 0.718, CI [−0.358, 2.456]), catastrophizing (*B_alternate_* = 0.662, SE = 0.713, CI [−0.736, 2.060]), or self-blame (*B_alternate_* = 0.974, SE = 0.678, CI [−0.355, 2.302]) were considered as predictors. Furthermore, none of the regulatory strategies predicted eating disorder symptoms when socially prescribed perfectionism, their corresponding latent interaction terms, and covariates were accounted for (*B_rumination_* = 0.274, SE = 0.859, CI [−1.410, 1.958]; *B_catastrophizing_* = 2.029, SE = 1.061, CI [−0.051, 4.109]); *B_self-blame_* = −2.337, SE = 2.283, CI [−6.812, 2.137]).

#### 3.2.2. Self-Oriented Perfectionism

Using a set of analyses similar to those conducted for socially prescribed perfectionism, we examined the moderating roles of rumination, catastrophizing, and self-blame strategies in the link between self-oriented perfectionism and eating disorder symptoms while controlling for covariates. We failed to find significant interaction effects between self-oriented perfectionism and the regulation strategies of rumination (*B* = 0.728, SE = 0.460, 95% CI [−0.173, 1.629]), catastrophizing (*B* = −1.151, SE = 0.1.091, 95% CI [−3.288, 0.987], or self-blame (*B* = −2.056, SE = 1.444, 95% CI [−4.886, 0.774). Log likelihood-ratio (Δ-2LL) tests showed that all baseline models did not differ from their corresponding alternate models, which included an interaction term, and thus, lends support to baseline models over alternate models.

Self-oriented perfectionism significantly predicted eating disorder symptoms in all baseline models in which rumination (*B_baseline_* = 0.271, SE = 0.077, 95% CI [0.776, 2.916]), catastrophizing (*B_baseline_* = 0.211, SE = 0.081, 95% CI [0.318, 2.478]), and self-blame (*B_baseline_* = 0.238, SE = 0.078, 95% CI [0.512, 2.698]) were included as predictors. This held across all alternate models when interaction terms with rumination (*B_alternate_* = 1.909, SE = 0.588, 95% CI [0.757, 3.062]), catastrophizing (*B_alternate_* = 1.484, SE = 0.589, 95% CI [0.330, 2.638]), and self-blame (*B_alternate_* = 0.789, SE = 0.275, 95% CI [0.250, 1.329]) were included. Of the three regulatory strategies, none emerged as a significant predictor of eating disorder symptoms in all baseline (*B_rumination_* = −0.026, SE = 0.096, 95% CI [−1.792, 1.364]; *B_catastrophizing_* = 0.163, SE = 0.103, 95% CI [−0.414, 3.866]; *B_self-blame_* = 0.048, SE = 0.098, 95% CI [−1.081, 1.806]) or alternate models (*B_rumination_* = −0.301, SE = 0.824, 95% CI [−1.916, 1.314]; *B_catastrophizing_* = 1.654, SE = 1.107, 95% CI [−0.516, 3.824]; *B_self-blame_* = 0.917, SE = 1.172, 95% CI [−1.380, 3.214]).

## 4. Discussion

We demonstrate that the relation between socially prescribed perfectionism and eating disorder symptoms in young females is more pronounced in those with greater ruminative tendencies. Specifically, we expand on prior studies by delineating how specific dimensions of perfectionism and cognitive regulatory strategies interact to predict disordered eating, hence offering insight into the intrapsychic processes underlying eating disorder symptoms. These findings underscore the crucial role of emotion regulation strategies, particularly rumination, in modulating the relationship between perfectionism and eating disorder symptoms and provide support for cognitive-behavioral models of eating disorders that include perfectionism as a central risk factor [4,5,8].

Four notable findings warrant further discussion. First, we provide empirical evidence that rumination, but not catastrophizing or self-blame, reinforces the positive relation between socially prescribed perfectionism and eating disorder symptoms. These findings corroborate evidence from prior studies [21,37,38] and meta-analyses [19,20] that the use of ruminative strategies is linked to symptoms of eating disorders. Moreover, previous studies examining similar interactional relations have drawn on Gratz and Roemer’s (2004) model of difficulties with emotion management to demonstrate the moderating role of emotion dysregulation, particularly limited access to adaptive regulatory strategies, in the relation between perfectionism and eating disorder symptoms in young adults [24,77]. Hence, our study advances the literature by identifying rumination as a specific regulatory strategy that interacts with socially prescribed perfectionism to account for eating disorder symptoms. Furthermore, this extends cognitive-behavioral models of eating pathology by implying that rumination modulates how perfectionistic cognitions surrounding the failure to meet socially prescribed standards [5,13] foster symptoms of eating disorder, such as weight-related concerns and dietary restraint.

These findings hold implications for intrapsychic cognitive processes involved in eating disorder symptoms. Given that socially prescribed perfectionism involves beliefs that self-worth is contingent on fulfilling externally imposed standards and garnering the approval of others [25], our results suggest that perceptions of inadequacy associated with eating disorder symptoms may stem from the pursuit of externally imposed standards of thinness, restriction, and discipline rather than standards of personal achievement [78,79]. Furthermore, ruminative tendencies likely serve a crucial role in prolonging and exacerbating thoughts and feelings that highlight an overvaluation of gaining acceptance from others, which in turn fosters eating disorder symptoms. As specified by our additional analyses, these intrapsychic processes underlie how the interaction effect between socially prescribed perfectionism and rumination uniquely predicts weight concerns and dietary restraint (e.g., caloric restriction/rules and fear of weight gain) in particular. Through these eating disorder symptoms, individuals likely derive a sense of control to mitigate feelings of helplessness and inferiority resulting from high levels of socially prescribed perfectionism in conjunction with ruminative tendencies [51]. Importantly, our findings show that rumination is a core emotion regulatory strategy to target, particularly for individuals with elevated levels of socially prescribed perfectionism. Together, this provides insights into the role of diverse regulatory strategies that can inform interventions for eating disorder symptoms, such as the evidence-based management of ruminative tendencies, particularly for individuals with heightened levels of socially prescribed perfectionism. In a related vein, a fruitful avenue for future research lies in examining the potentially protective role of adaptive cognitive strategies (e.g., positive reappraisal [19]) in alleviating eating disorder symptoms in perfectionistic individuals.

Second, it is noteworthy that socially prescribed perfectionism failed to account for eating disorder symptoms when rumination, catastrophizing, or self-blame were accounted for. Importantly, we showed that socially prescribed perfectionism did not predict eating disorder symptoms independently, but only when interacting with high levels of rumination. Overall, our study highlights the fact that although socially prescribed perfectionism is an important correlate of eating disorder symptoms, it may not uniquely explain the variance in disordered eating when we consider other risk factors, such as maladaptive regulatory strategies, particularly rumination. This is commensurate with recent findings (e.g., [11]) whereby perfectionism alone may not significantly account for eating-related clinical impairment when emotion dysregulation is accounted for, and thus, draws attention to the interactional relations between perfectionism and emotion dysregulation. More broadly, this underscores the need to examine the effects of distinct dimensions of perfectionism in tandem with a wider range of pertinent risk factors to more comprehensively explain eating disorder symptoms. Furthermore, our findings intriguingly suggest that self-oriented—compared to socially prescribed—perfectionism may more robustly predict eating disorder symptoms when regulatory strategies and corresponding latent interaction terms are considered. Hence, this also calls attention to the importance of delineating between perfectionism dimensions when examining the interactive effects between perfectionism and other risk factors on eating disorders, especially given that self-oriented and socially prescribed perfectionism may be differentially related to specific categories of eating disorder symptoms, such as dieting, food preoccupation, and oral control (see [80]).

Our third notable finding is that catastrophizing independently predicted eating disorder symptoms, above and beyond the effects of socially prescribed perfectionism. Given that prior studies have reported higher levels of catastrophic worry in individuals diagnosed with an eating disorder compared to healthy controls (e.g., [22,81]), our study offers crucial evidence of the link between catastrophizing and a wider range of eating disorder symptomatology in nonclinical young women. Moreover, this reinforces theoretical accounts which postulate that symptoms of eating disorders (e.g., dietary restraint) function to maladaptively avoid aversive emotional states stemming from anxiety or worrying [82]. Specifically, our findings suggest that catastrophizing may, in part, be a perseverative cognitive process that maintains preoccupation with concerns about weight, shape, and eating. This likely triggers symptoms of eating disorders to enable escape from aversive thoughts and emotions and seek out a sense of safety [52,83]. In contrast, our study does not elucidate self-blame as an important correlate of eating disorder symptomatology in young female adults, which deviates from studies that highlight the role of self-blame in eating disorder symptoms and remission [17,39]. Drawing on the findings of Mantilla and Birgegård (2015), it is likely that self-blame may be more robustly related to eating disorder symptoms in clinical, help-seeking adolescent samples rather than nonclinical samples of young adults. Further empirical efforts are warranted to ascertain the psychological mechanisms that underlie how catastrophizing is related to eating disorder symptoms in community samples.

Our study is not without limitations. First, our cross-sectional design and lack of experimental controls limit causal inferences. In light of our cross-sectional findings, it would be fruitful for further research to utilize longitudinal designs or experimental manipulations to ascertain the interactive relations between perfectionism and emotion regulation strategies in predicting eating disorder symptoms in both clinical and nonclinical populations. Indeed, prior studies have successfully manipulated multidimensional perfectionism [84,85] in relation to restrictive eating in nonclinical samples, while other studies have induced self-focused rumination [86,87]. Second, our sample of female college students limits the generalizability of these findings to male adults who have been shown to exhibit eating disorder symptoms such as body image disturbances and muscularity concerns [88,89,90]. Third, although the temporal stability and validity of the EDE-Q is well-established [91], the clinically derived four-factor structure of EDE-Q has received limited empirical support (see [92,93]); numerous studies find poor separability of shape and weight concerns and alternative configurations in organizing eating concerns, dietary restraint, and shape/weight preoccupation [94,95,96]. Given the lack of consensus regarding a recommended factor structure for EDE-Q, it would be valuable for further research to examine the interactional effects of psychosocial risk factors in relation to alternative two- (e.g., [94]), three- (e.g., [97]), or four-factor (e.g., [98]) structures which may better explain specific populations’ eating disorder symptoms [see 91]. Moreover, the EDE-Q may not clearly distinguish between subclinical and pathological levels of disordered eating. The mean EDE-Q scores of our sample fell below those reported by clinical samples of eating disorder patients [99,100,101], thus indicating that our sample’s eating disorder symptoms likely fall below clinical threshold. Therefore, it would be valuable for future research to examine whether interactive relations between perfectionism and rumination manifest differently between nonclinical samples and patients with eating disorders. Fourth, while we accounted for comprehensive demographic, affective, and personality covariates, future research would benefit from consideration of other characteristics, such as specific medical conditions (e.g., hypertension and diabetes) or pregnancy, which impose dietary restrictions and may, thus, heighten the salience of eating-/shape-/weight-related concerns associated with eating disorders. Furthermore, although our findings support the cognitive regulatory strategy of rumination as a critical moderator in the link between socially prescribed perfectionism and eating disorder symptoms, it would be illuminating for future research to employ rumination measures specific to features of eating disorders (e.g., the ruminative response scale for eating disorders [102]) to determine whether the content of rumination (e.g., concerns related to the body in particular) exacerbates the effect of perfectionism on eating disorder symptoms. Finally, in light of the significant relations among eating disorder symptoms and indices of affective well-being including depression and anxiety symptoms (see Table 2), future studies should investigate the interactional relations between perfectionism and emotion regulation in relation to a wider scope of outcomes such as academic, physiological, and social functioning.

## 5. Conclusions

In sum, we found that the relation between socially prescribed perfectionism and eating disorder symptoms is reinforced when rumination strategies are employed. Our findings suggest that curbing ruminative tendencies may be conducive to attenuating eating disorder symptoms, particularly in young female adults with heightened levels of perfectionism. Furthermore, our findings call attention to the role of catastrophizing as a significant correlate of eating disorder symptoms. Together, our study underscores the need to examine a wider scope of potential risk factors beyond perfectionism, and to delineate different dimensions of perfectionism. In light of the potentially grave consequences of eating disorder symptomatology for psychological well-being and health outcomes, our findings advance theoretical understanding of symptom development and provide practical insights into psychosocial factors for early interventions that target young women.

## Figures and Tables

**Table 1:** Descriptive statistics of predictors, covariates, and criterion variables.

	*M*	*SD*	Min	Max	Skewness	Kurtosis	Reliability ^1^
**Criterion**							
Disordered eating							
Shape concerns	24.54	10.14	8.00	48	0.38	−0.81	0.86
Weight concerns	12.56	6.00	5.00	27	0.64	−0.59	0.84
Eating concerns	9.91	5.57	5.00	31	1.71	2.91	0.78
Restraint	12.69	6.76	4.00	28	0.62	−0.62	0.82
**Focal Predictors**							0.77
Socially prescribed perfectionism	57.26	10.05	33.00	85.00	−0.04	−0.16	0.77
Self-oriented perfectionism	68.64	13.15	35.00	98.00	−0.11	−0.60	0.88
**Moderators**							
Rumination	14.16	2.86	7.00	20.00	−0.19	−0.25	0.68
Catastrophizing	9.72	3.43	4.00	18.00	0.36	−0.68	0.81
Self-blame	12.35	3.10	7.00	20.00	0.10	−0.78	0.80
**Covariates**							
Negative affect	22.88	8.00	10.00	49.00	0.657	0.248	0.90
Depressive symptoms	11.61	4.61	0	23.00	−0.068	0.222	0.89
Anxiety symptoms	11.41	4.64	0	24.00	0.151	0.465	0.87
Neuroticism	26.17	5.04	10.00	40.00	0.084	0.107	0.83
Conscientiousness	29.43	4.75	11.00	37.00	−0.775	0.943	0.72
**Demographics**							
Age (years)	21.6	0.52	19.00	27	2.39	3.93	-
BMI	20.69	2.56	15.35	30.12	0.55	0.61	-
Household income ^2^	2.43	1.47	1.00	6.00	1.12	0.45	-

*Note*. ^1^ Reliability estimates were computed based on Cronbach’s alpha. ^2^ Combined monthly income was rated on a 6-point scale (1 = below $5000; 2 = $5000–$10,000; 3 = $11,000–$25,000; 4 = $26,000–$50,000; 5 = $51,000–$100,000; 6 = above $100,000).

**Table 2:** Zero-order correlations between variables of interest.

	1	2	3	4	5	6	7	8	9	10	11	12	13	14	15	16	17
1. SPP	-	-	-	-													
2. SOP	**0.36**	-	-	-													
3. Rumination	**0.19**	**0.26**	-	-													
4. Catastrophizing	**0.25**	**0.19**	**0.36**	-													
5. Self-blame	**0.16**	0.15	**0.52**	**0.28**	-												
6. Global eating	**0.21**	**0.17**	**0.20**	**0.20**	0.12	-											
7. Shape concerns	**0.21**	**0.21**	**0.23**	**0.20**	**0.17**	**0.95**	-										
8. Weight concerns	**0.25**	**0.20**	**0.20**	**0.24**	0.11	**0.93**	**0.90**	-									
9. Eating concerns	0.12	0.02	0.09	0.14	0.07	**0.84**	**0.70**	**0.70**	-								
10. Restraint	0.14	0.14	**0.17**	0.08	0.00	**0.77**	**0.63**	**0.61**	**0.59**	-							
11. Negative affect	**0.17**	0.11	**0.36**	**0.44**	**0.25**	**0.20**	**0.22**	**0.19**	**0.16**	0.12	-						
12. Depressive symptoms	0.09	0.15	**0.29**	**0.45**	**0.23**	**0.33**	**0.35**	**0.34**	**0.31**	0.13	**0.44**	-					
13. Anxiety symptoms	0.03	0.04	**0.22**	**0.42**	**0.18**	**0.38**	**0.38**	**0.35**	**0.38**	**0.17**	**0.53**	**0.80**	-				
14. Neuroticism	0.04	0.09	**0.41**	**0.36**	**0.27**	**0.21**	**0.25**	**0.17**	0.12	0.15	**0.46**	**0.47**	**0.44**	-			
15. Conscientiousness	**−0.17**	**0.28**	**−0.18**	−0.11	−0.13	**−0.06**	**−0.11**	0.00	−0.11	0.04	**−0.18**	**−0.16**	−0.09	**−0** **.23**	-		
16. Age	**0.32**	−0.10	0.11	−0.06	0.14	−0.11	−0.09	−0.10	−0.13	−0.06	0.05	**−0.45**	**−0** **.47**	0.00	**−0** **.24**	-	
17. BMI	0.01	0.01	**0.18**	0.09	0.15	**0.40**	**0.39**	**0.43**	**0.28**	**0.26**	**0.19**	0.10	0.15	0.11	−0.02	0.10	-
18. Income	−0.01	−0.14	−0.13	0.00	−0.11	0.01	−0.04	0.03	0.07	0.02	0.04.	−0.07	0.01	−0.04	−0.01	−0.01	0.00

*Note.* Significant correlations marked in boldface. *p* < 0.05. SPP = socially prescribed perfectionism; SOP = self-oriented perfectionism.

**Table 3:** Fit indices for measurement and structural models.

	Χ^2^/*df*	RMSEA	SRMR	CFI	TLI	Log-Likelihood Ratio (Ho)
**Individual measurement models**						
Socially prescribed perfectionism (SPP)	0	0.000	0.000	1.000	1.000	-
Self-oriented perfectionism (SOP)	0	0.000	0.000	1.000	1.000	-
Rumination	0	0.000	0.002	1.000	1.000	-
Catastrophizing	0	0.000	0.010	1.000	1.000	-
Self-blame	0	0.000	0.006	1.000	1.000	-
Eating disorder (ED) symptoms	0	0.000	0.000	1.000	1.000	-
**Full-measurement models**						
SPP, ED symptoms, rumination	1.12	0.028	0.051	0.994	0.992	-
SPP, ED symptoms, catastrophizing	1.73	0.070	0.050	0.962	0.946	-
SPP, ED symptoms, self-blame	1.60	0.064	0.066	0.972	0.959	-
SOP, ED symptoms, rumination	1.37	0.050	0.053	0.984	0.978	-
SOP, ED symptoms, catastrophizing	2.07	0.085	0.062	0.954	0.933	-
SOP, ED symptoms, self-blame	1.46	0.056	0.065	0.981	0.973	-
**Structural models**						
Models with SPP as a focal predictor						
*Rumination*						
Baseline model	2.84	0.112	0.160	0.777	0.730	−2762.209
Alternate model ^1^	-	-	-	-	-	−2761.984
*Catastrophizing*						
Baseline model	2.70	0.107	0.107	0.791	0.749	−2749.096
Alternate model ^1^	-	-	-	-	-	−2747.615
*Self-blame*						
Baseline model	2.46	0.099	0.132	0.824	0.788	−2756.422
Alternate model ^1^	-	-	-	-	-	−2792.622
Models with SOP as a focal predictor						
*Rumination*						
Baseline model	3.12	0.120	0.160	0.780	0.731	−2694.445
Alternate model ^1^	-	-	-	-	-	−2695.438
*Catastrophizing*						
Baseline model ^1^	2.99	0.116	0.112	0.785	0.744	−2689.587
Alternate model	-	-	-	-	-	−2688.996
*Self-blame*						
Baseline model	3.52	0.131	0.140	0.734	0.684	−2737.771
Alternate model ^1^	-	-	-	-	-	−2736.300

*Note.* SPP = socially prescribed perfectionism; SOP = self-oriented perfectionism; RMSEA = root-mean-square error of approximation; SRMR = standardized root-mean-square residual; CFI = comparative fit index. ^1^ Alternate models include the respective latent interaction terms.

## Data Availability

Data and materials will be available upon request.
